# Are there any good experiments that should not be done?

**DOI:** 10.1371/journal.pbio.3001539

**Published:** 2022-02-14

**Authors:** Robert Pollack, Matthew Cobb

**Affiliations:** 1 Department of Biological Sciences, Columbia University, New York, New York, United States of America; 2 School of Biological Sciences, University of Manchester, Manchester, United Kingdom

## Abstract

Is there any research you think should not be done? Can you imagine deciding not to do an experiment? This Perspective article explores controversial experiments in the past and today, and what should be done.

## Teaser

Is there any research that should not be done? Could you think of an experiment and then decide not to do it? These questions get to the heart of the power of modern genetics to mix up and alter genes.

Over half a century ago, one of us (RP) was faced with a quandary. In June 1971, as an instructor on a Cold Spring Harbor Laboratory training course, Pollack learned of a proposed experiment in Paul Berg’s Stanford laboratory that aimed to introduce DNA from the SV40 virus, which causes cancer in hamster cells, into *E*. *coli*, which grows in the human gut. This would be the first ever such experiment, and Pollack was concerned that it might produce cancers in humans. The subsequent debate laid the ground for the 1975 Asilomar conference on recombinant DNA and the introduction of clear biosecurity measures that enable the safe use of this technology.

Together with the late Joseph Sambrook, Pollack drafted a letter to *Science* and *Nature* about the potential dangers of some recombinant DNA experiments (Box 1). The letter was never sent—the authors feared that Cold Spring Harbor Laboratory Director James Watson would retaliate in response to what he might see as criticism. Nevertheless, today’s students, postdocs, and junior staff should consider how they would respond if faced with a similarly worrying experiment.

**Box 1. Extract from a letter to the editors of *Nature* and *Science*, written by Robert Pollack and Robert Sambrook in June 1971, but never sent**

Are there any good experiments using human cells and viruses that should not be done?

Even if one chose to, one could not experiment on human beings. We accept, therefore, that a boundary exists past which we must indulge our curiosity.

Work with somatic human cells in culture has proceeded without any apparent hesitation, defining the inner or ‘do-able’ side of this boundary. For example, at the border today we find work on human-hybrid cell lines and heterokaryons, human tumor cells and putative human tumor viruses, extension of the host-range of avian and murine TV to the human host cell, while well within it are experiments on detection of inborn errors of metabolism in cells in culture and on transplantation of human organs.

Now a class of experiments with human germ cells, in vitro fertilization, cloning, introduction of ‘absent’ genes, or gene therapy is becoming possible. Should those experiments be on the same side as experiments on skin-biopsy cells, or should they be cloned with experiments on people?

A second related class of experiments involves the reverse process: putting human genes or the nucleic acid of human viruses into cells of other species, or into prokaryotic cells. The dangers (e.g., of creating a tumor virus that can grow inside a bacteria like *E*. *coli* which normally sits in the human gut) are immense.

Before work continues on either of these two classes of experiments, we suggest a portion of any scientist’s right to follow his (sic) nose without regard to consequences should be surrendered. We ought to ask ourselves whether the experimental results are worth the calculable and unknown dangers to ourselves and to the general population.

We propose that in this field if no other, we are obliged to ask ourselves whether the experiment needs to be done, rather than if it ought to be done, or if it can be done. If it is dangerous, or wrong, or both, and if it doesn’t need to be done, we just ought not to do it.

Such experiments are called “dual use research of concern” (DURC)—legitimate research that could be transformed into an offensive capability, or which entails a massive risk. The dangers are very real. In 2011, two “gain-of-function” studies of the H5N1 “bird flu” virus deliberately enabled the virus to be transmitted by aerosol or respiratory droplets [1,2]. Profoundly alarmed, 40 virologists adopted an immediate 60-day pause on H5N1 gain-of-function research while better safety protocols were adopted [3].

This was the only time since Asilomar that scientists have decided not to do potentially dangerous experiments. But, as at Asilomar, the scientists’ concerns were purely technical and safety oriented—there was no real challenge to the legitimacy of such studies.

Because there is no overall authority for deciding if such research is safe and needed, the problems soon resurfaced. In June 2014, US and Japanese scientists used avian flu sequences with high homology to the 1918 flu pandemic virus to create a new virulent virus. Later that year, at a time of general concern about potential pandemics, the US government imposed a moratorium on funding gain-of-function experiments in influenza, SARS, or MERS viruses. That ban was rescinded in January 2017 [4]. In 2016, Canadian scientists reconstructed the horsepox virus—closely related to smallpox—with the implication that the only human disease we have eradicated could be resuscitated [5].

At the moment, only 5% of countries regulate DURC; nowhere is such regulation clearly effective [6]. Between 2004 and 2010, there were 11 instances of infection in US labs through inadvertent pathogen exposure [7]. In 2014, 84 Centers for Disease Control (CDC) workers were potentially exposed to anthrax when live samples were accidentally distributed to 3 laboratories, while another CDC laboratory contaminated a flu sample with H5N1 and then shipped it to a government facility. In 2012, there were more than 2 possible release or loss events every week in US laboratories working with the most dangerous pathogens [8].

Not all alarming experiments involve pathogens. In 2013 and 2014, researchers at MIT and in the University of California at San Diego separately used CRISPR to make a “gene drive.” These homing endonuclease genes exponentially increase their frequency, and when linked to genes involved in reproduction could lead to the extinction or transformation of a local pest population. As well as the potential for pest control, both groups realized the danger of massive ecological disruption—this was a kind of genetic bomb. MIT PhD student Kevin Esvelt kept the discovery to himself for a month and did not even tell his supervisor; the UCSD group considered not publishing.

In 2014, Jennifer Doudna became disturbed when she heard a researcher describe using an airborne virus to carry CRISPR components into mice to create a model of human lung cancer, simply by breathing [9]. As she said to a reporter, “It seemed incredibly scary that you might have students who were working with such a thing.” [10].

Some experiments are too important to be left to the scientists. Genetic research provides 3 striking examples. The CRISPR babies scandal, which saw 3 healthy embryos mutated by Dr. He Jiankui, led to a global outcry, but there is no agreement on whether there should even be a moratorium on such research [11]. Indeed, before the scandal broke, the scientific community claimed to be charting a “prudent path” to human genome editing, dropping initial calls for a global consensus on the matter [12]. Gain-of-function pathogen research requires exhaustive justifications to DURC committees and funders, but there is no involvement of the global public in these debates. Finally, although researchers involved in developing gene drives have expressed their concerns about this technology, creating a global regulatory agreement will be difficult [13].

These are not simply questions of safety; the underlying usefulness of the experiments must also be addressed. In each of these examples, a case can be made that the real risks far outweigh the potential benefits and that these kinds of experiments should not be performed, either through self-restraint or via a global legal framework. Researchers need to take responsibility for the consequences of their science. Fifty years on, the question remains: Are there any good experiments we should not do?
